# Account of Diseases in an Eastern District of London

**Published:** 1802-07

**Authors:** 


### ACCOUNT OF DISEASES IN AN EASTERN DISTRICT OF LONDON,

*From May 20, to June 20, 1802.*

| | |
|---|---|
| **ACUTE DISEASES.** | |
| Febris Intermittens Tertiana | 5 |
| ———— Quotid. | 1 |
| Pneumonia - - - - - - | 3 |
| Catarrhus - - - - - - | 4 |
| Scarlatina Anginofa - - - | 3 |
| Cynanche Tonfillaris - - | 4 |
| Rheumatifmus Acutus - - | 2 |
| **CHRONIC DISEASES.** | |
| Tuffis - - - - - - - - | 10 |
| Dyfpnœa - - - - - - - | 12 |
| Tuffis cum Dyfpnœa - - | 10 |
| Phthifis Pulmonalis - - - | 4 |
| Afthma - - - - - - - | 1 |
| Hydrothorax - - - - - | 3 |
| Palpitatio Cordis - - - | 1 |
| Anafarca - - - - - - - | 5 |
| Afcites - - - - - - - | 3 |
| Syncope - - - - - - - | 1 |
| Epilepfia - - - - - - - | 3 |
| Paralyfis - - - - - - - | 4 |
| Cephalalgia - - - - - - | 8 |
| Gaftrodynia - - - - - - | 10 |
| Hæmorrhois - - - - - - | 5 |
| Scrophula - - - - - - - | 5 |
| Herpes - - - - - - - | 7 |
| Rheumatifmus Chronicus - | 16 |
| **PUERPERAL DISEASES.** | |
| Ephemera - - - - - - - | 3 |
| Menorrhagia Lochialis - - | 4 |
| Peritonitis - - - - - - | 2 |
| **INFANTILE DISEASES.** | |
| Rubeola - - - - - - - | 2 |
| Pertuffis - - - - - - - | 7 |
| Vermes - - - - - - - | 2 |
| Herpes - - - - - - - | 5 |
| Eryfipelas Infantile - - - | 1 |

Far as the feafon of the year has advanced beyond that period in which coughs, colds, and different difeafes of the pneumonic kind ufually appear, we have, till very lately, feen a number of patients labouring under thefe complaints. The long continuance of cold winds have undoubtedly been the occafion of prolonging the appearance of thefe difeafes beyond the ufual term. The change in the ftate of the weather which has now taken place has diminifhed the number of thefe cafes, and the fymptoms of thefe difeafes appear in a milder form.

The difeafes of children, which have been fo frequently taken notice of in thefe late Reports, have declined in the number of cafes which have occurred, and in the degree of aggravation with which the different fymptoms have been attended. The meafles and hooping cough, however, are not yet extinct, though they form a lefs prominent feature in the general appearance of difeafe. The throat has lately been a frequent feat of difeafe both in children and adults. Frequent inftances of the fcarlatina anginofa have occurred. This difeafe has in many cafes been attended with its ufual fymptoms, and without any peculiar degree of aggravation. The tonfils have been fwelled and inflamed, and confequently a degree of pain has been felt upon deglutition; a few white floughs have appeared, which have not, however, been fucceeded by any troublefome ulcers; the fever has fubfided under the ufe of common means, and and the eruption has gradually difappeared. Cafes, however, of a different defcription might be felected from the Reports of medical practitioners, in which fymptoms of the moft alarming nature appeared, and a fatal termination, in fome inftances, very fpeedily enfued. In one of the cafes of cynanche tonfillaris, befides the ufual fymptoms of rednefs and tumour of the fauces, and particularly of the tonfils, with difficult and painful deglutition and continued fever, there were fome apthous ulceration about the roof of the mouth, the tongue, the gums, and the infide of the cheeks. After fome days an eruption appeared on the hands, attended with fome degree of fwelling; upon which appearance, the fever gradually fubfided, the painful fymptoms in the throat abated, and in a fhort time the whole difeafe difappeared within the courfe of a few weeks. It has fallen to the lot of the prefent Reporter to have an unufual number of intermittent fevers under his care. In a converfation with fome other medical practitioners, he found that feveral cafes of a fimilar kind had fallen under their notice. Thefe intermittents have been chiefly of the tertian kind, and have yielded in general to the ufual mode of treatment.

